# Comparative Evaluation of the Stiffness of Abaca-Fiber-Reinforced Bio-Polyethylene and High Density Polyethylene Composites

**DOI:** 10.3390/polym15051096

**Published:** 2023-02-22

**Authors:** Faust Seculi, Francesc X. Espinach, Fernando Julián, Marc Delgado-Aguilar, Pere Mutjé, Quim Tarrés

**Affiliations:** LEPAMAP-PRODIS Research Group, University of Girona, 17003 Girona, Spain

**Keywords:** biopolymers, natural fibers, micromechanics, stiffness, intrinsic properties

## Abstract

The use of bio-based matrices together with natural fibers as reinforcement is a strategy for obtaining materials with competitive mechanical properties, costs, and environmental impacts. However, bio-based matrices, unknown by the industry, can be a market entry barrier. The use of bio-polyethylene, which has properties similar to polyethylene, can overcome that barrier. In this study, composites reinforced with abaca fibers used as reinforcement for bio-polyethylene and high density polyethylene are prepared and tensile tested. A micromechanics analysis is deployed to measure the contributions of the matrices and reinforcements and to measure the evolution of these contributions regarding AF content and matrix nature. The results show that the mechanical properties of the composites with bio-polyethylene as a matrix were slightly higher than those of the composites with polyethylene as a matrix. It was also found that the contribution of the fibers to the Young’s moduli of the composites was susceptible to the percentage of reinforcement and the nature of the matrices. The results show that it is possible to obtain fully bio-based composites with mechanical properties similar to those of partially bio-based polyolefin or even some forms of glass fiber-reinforced polyolefin.

## 1. Introduction

Composite materials are expected to have enhanced properties with respect to their matrices, and such properties are a combination of the properties of their phases [1,2]. These properties will contribute to the competitiveness of new composite materials compared to known or commercial materials, and can be measured in terms of mechanical or chemical properties, costs, or environmental impacts, among others [3,4,5]. In the case of natural fiber-reinforced composites, the mechanical properties of the matrix are enhanced by adding a more rigid phase in the form of adding more or less individualized fibers. By adding such fibers, the composites are expected to have higher tensile and flexural properties than the matrix has [6,7,8,9]. Cost competitiveness can be realized when the cost of the composites is cheaper than the materials used to replace them or when the ratio between the properties and the cost of the composite facilitate its adoption [10,11]. An example of a property that could allow the replacement of a more expensive material with a cheaper one is lightness, which can reduce energy expenditure when it is present in vehicles [12]. The environmental impact of a material has to be measured using scientific methods like life cycle analysis to assess its footprint on the environment [13]. In the case of composite materials, the main efforts have been devoted to replacing mineral reinforcements with natural fibers, and more recently, to replacing oil-based matrices with matrices based on renewable sources [14,15,16]. To do so, a complete knowledge of a polymer’s structure and processes is needed for its obtention, but this is not always available [17,18,19,20]. To facilitate property, cost, and environmental competitiveness, some researchers suggest the use of natural fibers with high intrinsic properties, the enhancement of the interface between these fibers and the continuous phase, and the use of bio-based matrices [21,22].

Polymer properties are widely characterized in the literature as having a large presence of polyolefin, but the characterization of biopolymers is less notable. The most present biopolymer is poly (lactic acid), and there is some research devoted to biobased polyamides (BioPA), polybutylene adipate terephthalate (PBAT), polycaprolactone, and starch polymers [23,24,25,26]. Polyethylene (PE) is a commonly used plastic, and high density polyethylene (HDPE) is the most present type of PE [27,28,29]. BioPE is a non-biodegradable plastic from biobased monomers [30]. BioPE made up 10.5% of the biopolymers market in 2019, which is only lower than PLA (18.7%), starch blends (18.7%), PBAT (13.5%), and, BioPAs (11.9%). BioPE is, along with BioPAs, one of the major players in the non-biodegradable bio-based plastic market [31]. One of the major advantages of BioPE is its similarities with HDPE [32,33]. These similarities include the fact that both allow the replacement of one polymer with the other with almost no changes to the equipment. In terms of costs, BioPE is more expensive than petrochemical PE. Moreover, the environmental footprint of BioPE is significantly lower than that of oil-based PE [32].

Regarding reinforcements, commercial composite materials are mainly reinforced with glass fibers (GF) [34,35]. These fibers, as manmade materials, have the morphological and property advantage of low scatter. Moreover, their intrinsic properties have high values and ensure noticeable increases in the tensile strengths and moduli of their composites [36]. Nonetheless, GF has some drawbacks: it is fragile, which limits the recyclability of its composites; it causes attrition to equipment, which decreases its durability; it is harmful when inhaled or when it comes into contact with the skin [37]. Natural fibers have been successfully used as a replacement for GF, obtaining competitive materials that tend to be used for automotive, aeronautical, or construction purposes [21,38,39,40,41]. The most well-known natural fibers with highly notable intrinsic properties are abaca, hemp, jute, sisal cotton, and linen [39,42,43]. Among them, abaca stands out due to its mechanical properties, including intrinsic tensile strengths ranging from 430 to 1135 MPa and intrinsic Young’s moduli ranging from 9.8 to 41 GPa [42,44,45]. The literature characterizes abaca fibers as difficult to decompose and as similar to GF in terms of their flexural properties [42,45]. However, abaca fibers are hydrophilic, and chemical treatments are needed to obtain composites with strong interfaces. In a recent article, the authors established that adding 8 wt.% of a coupling agent based on PE functionalized with maleic acid (MAPE) noticeably increased the tensile strengths of HDPE and BioPE-based composites reinforced with abaca fibers (AF) [46,47]. While it is possible to find relevant studies on the use of abaca fibers as reinforcement for oil-based composites, to the best of the authors’ knowledge, there are no studies on the stiffness of BioPE-based composites reinforced with such fibers [48,49,50].

Stiffness is the ability of a material to resist deformation when a load is applied. While Young’s modulus does not evaluate the stiffness of a material, both are proportional to each other. Therefore, stiff materials have high Young’s moduli, and flexible materials have low ones. Thus, Young’s modulus can be a good metric to use to comparatively evaluate the stiffness of materials. Regardless of the value of their Young’s moduli, materials cannot deform infinitely without breaking or sustaining plastic deformations. In the case of brittle materials—and this is also the case for natural fiber-reinforced composites—the plastic region is small compared to the elastic region, and a break occurs near the elastic limit. The comparison made between HDPE and BioPE composites will be based on these properties.

Natural fibers are more flexible than GF, and it is possible to add higher percentages of natural fibers to a composite than is possible with GF [51]. It is difficult to find GF-based materials that add more than 30 wt.% of reinforcement. This is an advantage of natural fibers in terms of the costs of the composites. Natural fibers are cheap compared to their matrices, and thus, the higher the amount of reinforcement, the cheaper the resulting composite [52,53]. The percentage of reinforcement is limited by a fiber’s viscosity, which makes its transformation difficult. Regardless, it is possible to find in the literature composite materials with 60 wt.% of natural fibers that can be mold injected or extruded [54,55].

In this study, composite materials with abaca fiber used as HDPE and BioPE reinforcements were formulated, mixed, and tensile tested. AF ranging from 20 to 50 wt.% was added to the composites. Concerning reinforcement content, 8 wt.% of MAPE was added to all composites. The evolution of the tensile properties of the composites (strength, Young’s modulus, and strain at the break) was presented and discussed. Micromechanical analysis, based on a modified rule of mixtures and Hirsch’s equation, was used to evaluate the contribution of the phases to the Young’s moduli of the composites, revealing that the factors contributing to the dispersed phase went from being oil-based composites to bio-based ones. The intrinsic Young’s modulus of AF was back-calculated, and its dependence on the fiber percentage and the nature of the matrix was exposed and discussed. The mean orientation of the fibers against the load direction and the packing distribution of the fibers was proposed. A second micromechanics model based on the Halpin, Tsai, and Pagano models was used to support the obtained results. The properties of bio-based composites were found to be better than those based on HDPE, indicating the possibility of replacing partially bio-based composites with fully bio-based ones. Moreover, BioPE-based composites could possibly replace polypropylene, HDPE, and some of its GF-based composites at 10 to 20 wt.% of AF content. Adding high amounts of AF could be a way to obtain materials with competitive properties and cost and also with reduced environmental impact.

## 2. Materials and Methods

### 2.1. Materials

Two different matrices were tested in a continuous phase for the composite materials. On the one hand, an oil-based HDPE (HDI0661U1) with a density of 0.953 and a melt flow index of 26 g/10 min (190 °C; 2.16 kg) was tested. On the other hand, a bio-based polyethylene (SHA7260) and a density of 0.955 g/cm^3^ and a melt flow index of 26 g/10 min (190 °C; 2.16 kg) was tested. Both matrices are commercially available from Braskem (Sao Paulo, Brasil) and have a density of 0.95 g/cm^3^. Following the findings of a recent article using these matrices, polyethylene functionalized with maleic acid (from here on MAPE) was used as a coupling agent to increase the strength of the interface between the matrix and the reinforcements [46]. This coupling agent commercially available from Dupont (Wilmington, DE, USA) and is referred to as Fusabond^®^ MB100D, 0.9%. The coupling agent has a density of 0.96 g/cm^3^, a melt flow index of 2.0 g/10 min (190 °C; 2.16 kg), and 0.55 wt.% of maleic acid [56].

The dispersed phase of the composites was made of abaca fibers from the Philippines kindly provided by CELESA (Tortosa, Spain). The composition of these fibers was analyzed in a previous publication [46].

The composites will from hereon be referred to with the following nomenclature: MATRIXnAFmMAPE, where MATRIX is HDPE or BioPE, n is the wt.% of AF content, and m is the MAPE’s wt.% content.

### 2.2. Composites Mixing and Tensile Test Specimen Mold Injection

A Brabender^®^ Plastograph mixer was used to compound the phases. The weight percentages were added to the equipment and the mixing was executed at 180 °C and 80 rpm for 10 min. These process parameters ensured the proper mixing of the phases and the homogeneous dispersion of the fibers in the matrix while avoiding excessive fiber shortening due to attrition phenomena [57,58]. The resulting composites were pelletized, after cooling down, in a hammer mill. This equipment produced pellets with a 5 mm mean diameter that were able to be used in the mold injection equipment. These pellets were dehumidified at 80 °C for 24 h before being mold injected. The equipment used for the mold injection was Meteor^®^ 40 (Mateu and Solé, Barcelona, Spain). The machine was operated at an injection pressure of 120 kg/cm^2^ and a maintenance pressure of 25 kg/cm^2^. The temperatures of the three heating areas of the equipment were 175 °C, 175 °C, and 190 °C.

### 2.3. Tensile Testing of the Composites

Before the tensile test, and in accordance with ASTM D638, specimens were stored for 48 h in a conditioning chamber. This chamber was operated at a relative humidity of 50% and at 23 °C. Tensile test facilities use a universal testing machine by Instron^®^, the product reference of which is 1122. The equipment was provided by Metrotec, S.A (Barcelona, Spain), and had a 5 kN load cell operating at 2 mm/min. Tensile tests were carried out in accordance with ISO 527-1:2000. All specimen widths and thicknesses were measured at three different points, and these measurements were used to compute the area perpendicular to the tensile load. The tensile properties were determined by the mean values of at least five experiments. An extensometer was used to measure specimen deformations.

The experimental results, tensile strengths, Young’s moduli, and tensile strains were analyzed under ANOVA with R^®^ (R Foundation, Vienna, Austria). The tests were performed with a 95% confidence rate.

### 2.4. Abaca Fiber Extraction from the Composite and Its Morphologic Characterization

AF was extracted from the HDPE by solubilizing the matrix with bicyclo [4.4.0]decane (decalin) in a Soxhlet apparatus. The specimens were cut into small pieces, and the pieces were put inside the Soxhlet equipment in a cellulose filter. Extraction was carried out for 24 h. Then, the extracted fibers were treated with acetone and distilled water to eliminate any solvent residue. Following this, the recovered fibers were stored in an oven at 105 °C for 24 h to eliminate humidity.

The morphology of the fibers was characterized in MorFi Compact (a morphological fiber analyzer), from Techpap SAS, (Gières, France). The fibers were introduced to the equipment, which analyzed 25,000 to 30,000 fibers and returned their length and diameter distribution, mean values, and also weighted values. The characterization was repeated four times for the reinforcements extracted from the composites, adding 20 to 50 wt.% AF content.

## 3. Results and Discussion

### 3.1. Evaluation of the Tensile Properties of the Composites

Table 1 shows the experimental values obtained from the tensile test on the AF-reinforced HDPE and BioPE composites.

**Figure 1:**
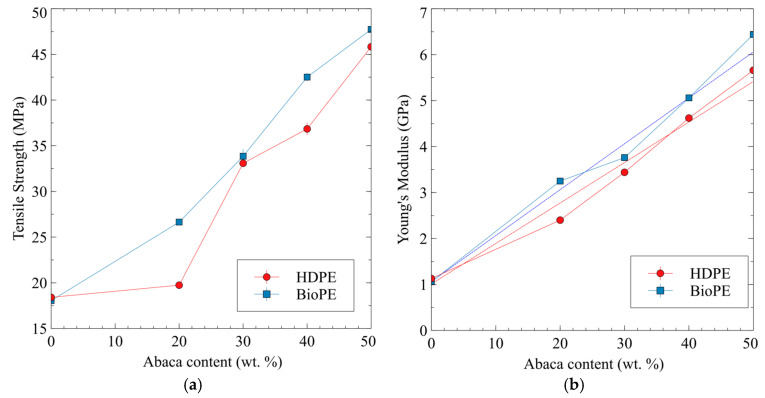
Evolution of the tensile properties of AF-reinforced HDPE and BioPE composites against abaca fiber contents; (**a**) tensile strength; (**b**) Young’s modulus.

The tensile properties increased with AF content for the HDPE and BioPE-based composites. The main difference was found for the materials with 20 wt.% AF content. While the HDPE-based materials showed a poor increase in their tensile strength concerning the matrix, the BioPE-based composites showed noticeable increases. It was found that the tensile strength of the HDPE20AF8MAPE composite was not statistically different from that of the matrix. Higher AF content returned materials with noticeably increased tensile strengths. Materials with 30 wt.% AF content showed similar tensile strengths, despite the matrix. BioPE-based composites with 40 and 50 wt.% AF content showed higher tensile strengths than the HDPE-based composites (Figure 1a). Thus, concerning tensile strength, the BioPE-based composites showed slightly higher tensile strengths than HDPE-based materials did. Taking into account that the tensile strengths of the matrices are statistically similar, the differences in those of BioPE-based materials may be due to the fact that the latter have a stronger interface than HDPE has.

The case of Young’s modulus is similar. Both matrices showed statistically similar Young’s moduli. Young’s modulus increased noticeably with AF content, despite the matrix. Figure 1b shows that the Young’s moduli of the BioPE-based composites were higher than those of the HDPE composites. The ANOVA analysis found that the values of only the composites with 30 wt.% of AF were statistically similar. The values for the composites with 20, 40, and 50 wt.% AF content were statistically different. Thus, in the case of stiffness, the BioPE had a greater competitive advantage over the HDPE-based materials. Moreover, for BioPE- and HDPE-based composites, the evolution of Young’s moduli against AF content was favorably linear, and this can be related to the fact that the reinforcements were well-dispersed in the composite materials [59].

HDPE and BioPE-based composites showed noticeable decreases in their strains at the break in relation to the amount of AF. The BioPE-based composites returned higher strains than HDPE did for with the same AF content. The strain at the break of any Bio-PE-based composite is statistically similar to the strain at the break of HDPE-based composites with 10% less AF content. Thus, the BioPE-based composites showed a higher ability to deform without breaking. This is another indicator of that a plasticizer can be used in the formulation of BioPE.

The results show that BioPE-based composites have similar tensile properties to HDPE-based materials. Thus, BioPE-based composites can be used to replace HDPE-based material under tensile loads. The ability of BioPE-based composites to replace other materials like glass fiber-reinforced polyolefin in terms of tensile properties can be evaluated by comparing the properties of such materials. The literature shows that HDPE-based composites reinforced with 20 and 30 wt.% of glass fiber achieved tensile strengths of 37.86 and 45.19 MPa, and Young’s moduli of 4.6 and 6 GPa, respectively. BioPE-based composite materials with 40 and 50 wt.% AF have tensile properties in this range (Table 1) [60,61,62,63]. Thus, BioPE-based composites can theoretically replace nonrenewable-based composites.

### 3.2. Contribution of the Phases to Young’s Modulus of the Composites

To evaluate the contribution of the phases to the Young’s modulus of the composites, the authors used the Fiber Tensile Modulus Factor (FTMF). This factor is based on a modified rule of mixtures for the Young’s modulus of short-fiber-reinforced composites (Equation (1)).

(1) $$E_{t}^{C}=\eta_{e}\times E_{t}^{F}\times V^{F}+\left(1-V^{F}\right)\times E_{t}^{M}$$

In the equation, $E_{t}^{C}$, $E_{t}^{F}$, and $E_{t}^{M}$ are Young’s moduli of the composite, reinforcement, and matrix, respectively. The volume fraction of the reinforcement is *V^F^*. A modulus efficiency factor, “$\eta_{e}$”, equalizes the stiffening efficiency of the fibers, taking into account the orientation and morphological properties of the reinforcement. This equation is a linear model, and can be used if the evolution of the property goes against that of the variable, in this case the reinforcement volume fraction. Figure 1b shows the behavior of the Young’s moduli of HDPE and BioPE composites, which can be fitted to a regression line, allowing the use of linear models. Most of the variables were obtained experimentally from the tensile test, except for the intrinsic Young’s modulus of the fibers and the modulus efficiency factor. Thus, Equation (1) has two unknown values and cannot be solved. Regardless, the contribution of the fibers is expected to be linear against its volume fraction and equal to $\eta_{e}\times E_{t}^{F}$. Thus, the authors evaluated the evolution of such contributions and computed the slope of the regression curve to obtain the FTMF. The contribution of the reinforcements was obtained by rearranging Equation (1) to Equation (2).

(2) $$\eta_{e}\times E_{t}^{F}=\frac{E_{t}^{C}-E_{t}^{M}\times \left(1-V^{F}\right)}{V^{F}}$$

**Figure 2:**
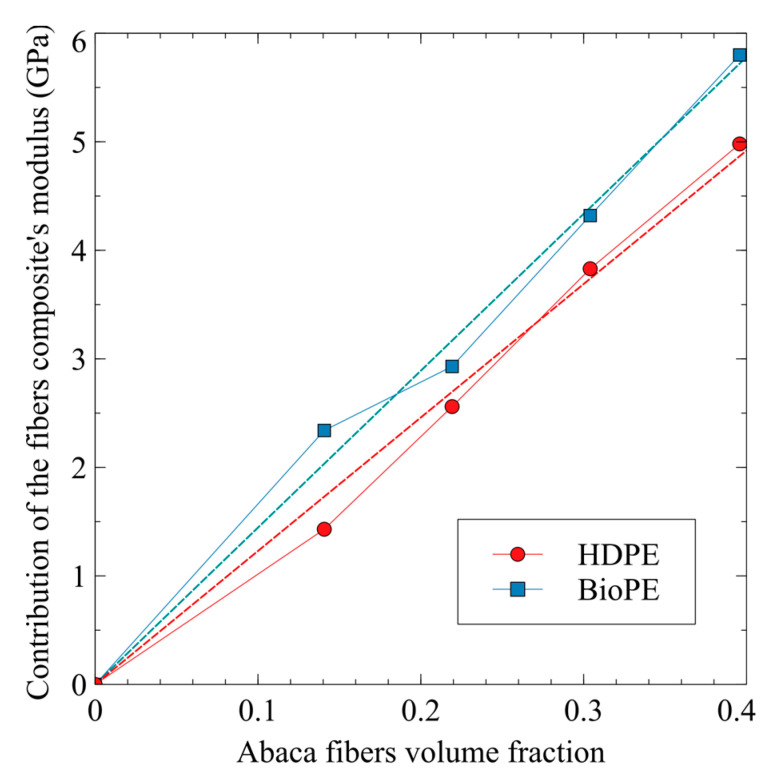
Contribution of the reinforcement phase to Young’s moduli of AF-reinforced HDPE and BioPE composites.

Figure 2 shows the evolution of the contributions of the fibers to the Young’s modulus of the composites against the AF content.

The slopes of the regression curves were found to be 12.3 and 14.5 for HDPE and BioPE-based composites, respectively. Taking into account that the fibers are the same, BioPE-based composites exploit the stiffening abilities of abaca fibers 15.2% more than HDPE-based composites do. Glass fibers used as polypropylene reinforcement returned a 32.7 FTMF [64]. Thus, abaca fibers as HDPE and BioPE reinforcement showed stiffening potentials that were 37.6% and 46.5% of GF’s, respectively. Hence, it will be necessary to use 62.4% and 53.5% of additional AF content for HDPE and BioPE composites, respectively, to obtain composites with the same Young’s moduli as GF-based ones. Adding higher amounts of natural fibers to a composite can positively impact its costs and its environmental impact. On the one hand, natural fibers have a noticeably lower cost than matrices do, and on the other hand, presumably, the environmental impact of natural fibers is expected to be lower than that of a matrix [11,65,66,67].

Using other natural fibers, namely alfa fibers, stone groundwood, old recycled newspaper fibers, and orange tree pruning fibers, returned 17.09, 10.33, 11.32, and 10.28 GPa FTMF‘s, respectively [62,64,68,69]. The stiffening potential of AF is lower than that of alfa fibers and superior to that of wood fibers, such as orange tree pruning fibers, recycled fibers from old newspapers, and stone groundwood fibers.

### 3.3. Micromechanics

Although the proposed modified rule of mixtures (Equation (1)) has two unknown values, the intrinsic Young’s moduli of the reinforcements can be obtained from micromechanical models like the Hirsh equation (Equation (3)) [70]:

(3) $$E_{t}^{C}=\beta \times (E_{t}^{F}\times V^{F}+E_{t}^{M}\left(1-V^{F})\right)+\left(1-\beta \right)\frac{E_{t}^{F}\times E_{t}^{M}}{E_{t}^{F}\times V^{F}+E_{t}^{M}\left(1-V^{F}\right)}$$

The Hirsch equation models Young’s modulus of a composite as the balance of the contributions of fibers aligned and perpendicular to the tensile loads. Aligned fibers (Voigt model) are impacted by a *β* parameter and perpendicular fibers (Reuss model) are impacted by 1 − *β* [71]. The literature indicates that a value of 0.4 *β* is appropriate for short fiber semi-aligned mold-injected composites [72]. Figure 1b shows that the evolution of the Young’s modulus of the composites depends on the volume fraction of the reinforcements, and while it is not completely linear, it can be approximated as a regression. This behavior allows using Hirsch equation.

Table 2 shows the obtained theoretical intrinsic moduli.

The values obtained for the intrinsic modulus of abaca fibers varied noticeably with the percentage of reinforcement and also with the nature of the matrix. This can be seen as anomalous as Young’s modulus of a material is a fundamental property and does not change unless the structure of the material changes. This is expected when such a property is obtained from a single fiber tensile test. Nonetheless, the values obtained from micromechanical models can vary from those of the intrinsic properties of the materials because micromechanical values are linked to the composite and to the exploitation of its potential for reinforcement [73]. In the case of the HDPE-based composite, the values showed the increasing exploitation of the stiffening potential of AF. On the other hand, the BioPE-based materials showed a descent in the exploitation of such potential. The mean values for the intrinsic Young’s moduli obtained for HDPE and BioPE-based composites are 26.57 ± 3.23 GPa and 29.26 ± 3.36 GPa, respectively. These values are statistically different with a 95% confidence rate and a *p*-value of 0.00021. Thus, this shows that the nature of the matrix impacted the exploitation of the stiffening potential of the fibers. This can be explained by the more homogeneous dispersion of the fibers inside BioPE than inside HDPE. This homogeneous dispersion prevents the presence of fiber bundles and allows the full exploitation of single fibers. The obtained values are consistent with the literature that provide values in a range from 9.8 GPa to 41 GPa [42,45]. Moreover, the model is based on the linear evolution of the Young’s moduli of the composites against the reinforcement volume fractions. While the evolution is quasi linear (Figure 1b), the deviations from linear behavior further facilitate the prediction of the intrinsic properties.

Once the intrinsic Young’s modulus of a fiber is known, Equation (1) can be used to obtain the value of the efficiency factor (Table 2). This factor indicates the exploitation of the stiffening potential of the fibers and is impacted mainly by the morphology of the fibers and their mean orientation angle. The literature shows that the expected values are between 0.4 and 0.7 [60,64,74]. The obtained values (Table 2) are in the low range, indicating that it can be possible to increase the exploitation of the fiber’s stiffening capabilities. The maximum value for the intrinsic Young’s modulus (38.13 GPa) can be used in Equation (1) for the composites with 50 wt.% AF. Then, HDPE and BioPE-based composites can reach the theoretical GPas of 7.47 and 7.43, respectively. A low efficiency factor of 0.45 was used to make the calculation. As commented above, the efficiency factor is impacted by the morphology of the fibers and their mean orientation, and can be expressed as the multiplication of the length efficiency factor ($\eta_{l}$) and the orientation factor ($\eta_{o}$), expressed as $\eta_{e}=\eta_{l}\times \eta_{o}$.

The length efficiency factor can be obtained from the Cox and Krenchel models (Equations (4) and (5)) [75]:

(4) ηl=1−tanh(γ×LF2)(γ×Lf2)

where

(5) γ=1RFEtMEtF×(1−ν)×lnπ4×VF

In the equations, the parameter γ reflects the stress concentrations at the end of the fibers, $R^{F}$ is the mean radius of the reinforcements, $L^{F}$ is their mean length, and ν is the Poisson’s ratio of the matrix (0.39). Mean lengths and diameters were extracted from the morphological analyses of the fibers extracted from the HDPE-based composites. The mean weighted lengths for composites with 20, 30, 40, and 50 wt.% AF were 1920, 1340, 1230, and 940 μm, respectively. The mean diameters for the same composites were 20.5, 19.5, 22.6, and 22.4 μm. Table 2 shows the obtained length efficiency factors.

For both HDPE and BioPE-based composites, the length efficiency factor is positively correlated with the percentage of fibers. This is linked to the effect of the viscosity of the composites on the mean length of the reinforcements [76]. As the percentage of fibers increases, the viscosity of the composites does the same, increasing the amount of attrition phenomena during mixing and decreasing the mean length of the reinforcements [76,77,78]. Length efficiency factors seem to be matrix-dependent because the values obtained for the HDPE and BioPE-based composites were mostly different with a 95% rate of confidence. Once the length efficiency and the length efficiency factors were known, the orientation efficiency factor could be obtained by the division of the known factors. Table 2 shows the computed values. The orientation efficiency factors were negatively correlated with the number of fibers. Thus, this implies that as the percentage of the fibers increases, the fibers tend to align with the load directions. This can be related to some phenomena. On the one hand, the decrease in the mean length of the fibers facilitates their alignment and decreases fiber bending. On the other hand, it is known that fibers in the skin region of the specimens tend to be aligned with the injection molding flow direction, while the fibers in the core region tend to show a random alignment [79]. Changes in the viscosity of the matrix can affect the dimensions of such regions, increasing the thickness of the skin region. The values for the orientation efficiency factors did not change noticeably with the nature of the matrices, and the composites with 30 and 40 wt.% AF content returned statistically equivalent orientation efficiency factors (Table 2).

These efficiency factors can be converted into limit angles and mean orientation angles thanks to the works of Fukuda and Kawata [80], and Sanomura and Kawamura [81]. Fukuda and Kawata provided three equations to compute a limit orientation angle depending on the fiber distribution inside the matrix. For a rectangular distribution, the authors provided Equation (6):

(6) $$\eta_{o}=\frac{sin\left(\alpha_{0}\right)}{\alpha_{0}}\left(\frac{3-\nu }{4}\frac{sin\left(\alpha_{0}\right)}{\alpha_{0}}+\frac{1-\nu }{4}\frac{sin\left(3\alpha_{0}\right)}{3\alpha_{0}}\right)$$

For a sinusoidal distribution, they provided Equation (7):

(7) $$\begin{array}{cc} \eta_{o} & =\frac{\pi^{2}}{16}\left(\frac{1}{\pi /2+\alpha_{0}}+\frac{1}{\pi /2-\alpha_{0}}\right)cos\left(\alpha_{0}\right)\\ & \times [\frac{3-\nu }{4}\left(\frac{1}{\pi /2+\alpha_{0}}+\frac{1}{\pi /2-\alpha_{0}}\right)\times cos\left(\alpha_{0}\right)\\ & +\frac{1+\nu }{4}\left(\frac{1}{\pi /2+3\alpha_{0}}+\frac{1}{\pi /2-3\alpha_{0}}\right)\times cos\left(3\alpha_{0}\right)] \end{array}$$

For a triangular distribution, they provided Equation (8):

(8) $$\eta_{o}=4\frac{1-cos\left(\alpha_{0}\right)}{\alpha_{0}^{2}}\left(\frac{3-\nu }{4}\frac{1-cos\left(\alpha_{0}\right)}{\alpha_{0}^{2}}+\frac{1+\nu }{4}\frac{1-cos\left(3\alpha_{0}\right)}{9\alpha_{0}^{2}}\right)$$

The limit angles of the rectangular, sinusoidal, and triangular distributions were obtained from these equations. In the equations, $\alpha_{0}$ is the limit angle.

Then, Sanomura and Kawamura provided the equation used to compute a mean orientation angle (Equation (9)):

(9) $$f_{p}=\frac{sin\left(2\alpha_{0}\right)}{2\alpha_{0}}=2cos^{2\;}\left(\alpha \right)-1$$

In the equation, $f_{p}$ is an orientation parameter that relates the limit angle to the mean orientation angle (α).

Table 3 shows the mean orientation angles obtained for the rectangular (*α*_r_), sinusoidal (*α*_s_), and triangular (*α*_t_) distributions.

Mean orientation angles indicate the deviation of the fibers from an aligned orientation corresponding to 0°. As expected, the mean orientation angles positively correlated with the orientation efficiency factor and the change in value depending on the fiber distribution. To select the fiber distribution that would better indicate the behavior of the composite, the authors considered the relation of the orientation factor (*χ*_1_), defined for its use in a modified Kelly and Tyson equation, to the mean orientation angle [82]. The literature shows that the orientation factors for semi-aligned short-fiber-reinforced mold-injected composites are usually in the range from 0.25 to 0.35 [83], and relates such factors with a mean orientation angle through:

(10) $$\chi_{1}=cos^{4}\left(\alpha \right)$$

Then, the expected mean orientation angles are between 39.7° and 45.0°. The distribution that shows mean orientation angles inside the proposed range is triangular. Thus, to simulate the behavior of the composite materials by finite elements, the authors suggest using this form of fiber distribution. These mean orientation angles correspond to 45.0% and 44.7% of the oriented fibers in the case of HDPE and BioPE-based composites, respectively. This is not far from the figure of 37.5% proposed by Tsai and Pagano [84] in their model to predict Young’s modulus of a composite (Equation (11)):

(11) $$E_{t}^{C}=\frac{3}{8}E^{11}+\frac{5}{8}E^{22}$$

*E*^11^ and *E*^22^ are the longitudinal and transversal moduli of the composite computed through the Halpin and Tsai equations:

(12) $$E^{11}=\frac{1+2\left(L^{F}/2R^{F}\right)\xi_{l}V^{F}}{1-\xi_{l}V^{F}}E_{t}^{M}$$

with

(13) $$\xi_{l}=\frac{\left(E_{t}^{F}/E_{t}^{M}\right)-1}{\left(E_{t}^{F}/E_{t}^{M}\right)+2\left(L^{F}/2R^{F}\right)}$$

and

(14) $$E^{22}=\frac{1+2\xi_{t}V^{F}}{1-\xi_{t}V^{F}}E_{t}^{M}$$

with

(15) $$\xi_{t}=t\frac{\left(E_{t}^{F}/E_{t}^{M}\right)-1}{\left(E_{t}^{F}/E_{t}^{M}\right)+2}$$

All the parameters present in the equation have been already defined.

**Figure 3:**
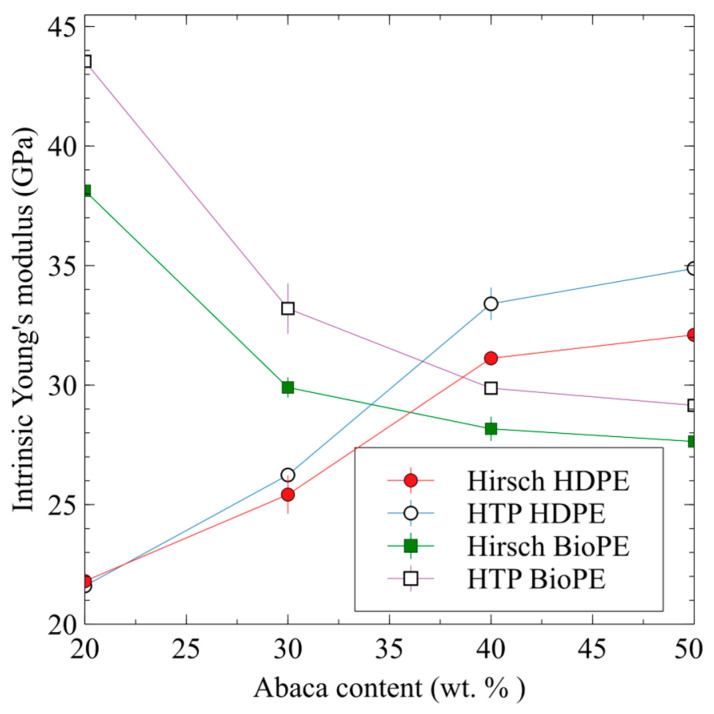
Abaca fiber’s intrinsic tensile Young’s modulus obtained from micromechanical models against fiber contents.

Table 3 shows the obtained results. Differing from the Hirsh equation (Equation (3)), the Tsai and Pagano model and the Halpin and Tsai equations (HTP) implicitly include the morphology of the fibers. The obtained results, like those obtained with Hirsch’s equation, show differences in the matrix and the fiber contents. Values obtained by using HTP returned higher intrinsic Young’s moduli than those obtained with Hirsch’s equation (Figure 3).

The values obtained from both methods showed the same tendencies. The intrinsic Young’s modulus was positively correlated with the fiber content in the case of the HDPE-based composites, and was negatively correlated with the content in the BioPE-based ones. In the case of the BioPE20AF8MAPE composite, the value returned by HTP was from the intrinsic Young’s moduli found in the literature and can be considered an overestimation. In any case, values obtained from HTP can be used to establish a range of theoretical values for the intrinsic Young’s modulus of AF. The results corroborate the differences between experimental intrinsic Young’s moduli and back-calculated ones [73,85]. These differences can be attributed to the assumptions of the models based on the rules of mixtures. Micromechanical models assume that the properties of the fibers are homogeneous all along the composite, and this is not true for natural fibers [86]. Moreover, the intrinsic properties of the fibers can be affected by the processes used during the manufacture of the composites, which shorten or damage these fibers [87].

## 4. Conclusions

Composites based on HDPE and BioPE reinforced with abaca fibers were mixed and tensile tested. The tensile strengths of the composites were positively correlated with the percentages of reinforcement. Composites based on the BioPE matrix showed slightly higher but similar tensile strengths to those based on HDPE when the AF content was equal or to higher than 40 wt.%. In terms of tensile strength, the BioPE-based composites showed the ability to replace oil-based materials. BioPE50AF8MAPE had a tensile strength that was 4% higher than that of HDPE50AF8MAPE.

The Young’s moduli of the composites were positively correlated with their AF content. The BioPE-based composites showed statistically significative higher values than the HDPE-based materials did. The BioPE50AF8MAPE had a Young’s modulus that was 14% higher than that of the HDPE50AF8MAPE and five times higher than that of the BioPE matrix.

The strains at the break were negatively correlated with AF content. The BioPE-based composites had a higher ability to deform without breaking. The BioPE composites showed strains at the break that were statistically similar to those of HDPE-based composites with 10 wt.% less AF.

Based on the values obtained for the fiber tensile modulus factor, to obtain a material with the same Young’s modulus as a GF-reinforced composite, the percentages of AF have to be theoretically 37.7% and 46.5% higher than that of GF for BioPE and HDPE-based materials. This can positively impact the cost and environmental impact of such materials. More research is needed to fully support this conclusion.

The intrinsic Young’s moduli for AF, obtained using the Hirsch equation, ranged from 21.8 GPa to 38.1 GPa.

The analyses of the mean orientation angle and the packing distribution showed that the most probable distribution was a triangular distribution with a mean orientation angle between 40.3° and 40.5°.

Future work should include the characterization of the composites by using a scanning electron microscope to assess the mechanics of the fractures. Thermal analyses are also needed to explore the impact of the fibers on thermal properties and on possible changes in the crystallinity of the polymers due to the presence of nucleating agents.

## Figures and Tables

**Table 1:** Influence of AF contents over the tensile strength ($\sigma_{t}^{C}$), the Young’s Modulus ($E_{t}^{C}$) and the strain at breaking ($\varepsilon_{t}^{C}$) of 8wt.% coupled composites.

Composite	$\sigma_{t}^{C}$ (MPa)	$E_{t}^{C}$ (GPa)	$\varepsilon_{t}^{C}$ (%)
HDPE	18.41 ± 0.07 ^a b^	1.13 ± 0.05 ^a^	8.61 ± 0.15 ^f^
HDPE20AF8MAPE	19.74 ± 0.12 ^b^	2.40 ± 0.08 ^i^	5.10 ± 0.32 ^d^
HDPE30AF8MAPE	33.08 ± 0.31 ^c^	3.44 ± 0.07 ^d c^	3.40 ± 0.41 ^c^
HDPE40AF8MAPE	36.84 ± 0.68 ^d^	4.62 ± 0.06 ^e^	2.75 ± 0.39 ^b^
HDPE50AF8MAPE	45.83 ± 1.20 ^f^	5.66 ± 0.09 ^g^	1.56 ± 0.19 ^a^
BioPE	18.05 ± 0.17 ^a^	1.06 ± 0.01 ^a^	9.67 ± 0.27 ^g^
BioPE20AF8MAPE	26.64 ± 0.24 ^e^	3.25 ± 0.03 ^b^	6.10 ± 0.29 ^e^
BioPE30AF8MAPE	33.85 ± 0.77 ^c^	3.76 ± 0.04 ^d^	4.86 ± 0.20 ^d^
BioPE40AF8MAPE	42.51 ± 0.45 ^g^	5.06 ± 0.01 ^f^	3.82 ± 0.26 ^c^
BioPE50AF8MAPE	47.73 ± 0.27 ^h^	6.44 ± 0.11 ^h^	2.7 ± 0.13 ^b^

Different letters, a, b, c, d, e, f, g, h and i, represent the statistical difference (ANOVA, *p* < 0.05) between the properties of the materials.

**Table 2:** Influence of AF content on the intrinsic Young’s modulus ($E_{t}^{F}$) and efficiency (*η_e_*), length (*η_l_*) and orientation (*η_o_*) factors of 8wt.% coupled composites.

Composite	$E_{t}^{F}$ (GPa)	*η_e_*	*η_l_*	*η_o_*
HDPE20AF8MAPE	21.80 ± 1.55 ^a^	0.465 ± 0.004 ^e^	0.810 ± 0.007 ^b^	0.574 ± 0.001 ^e^
HDPE30AF8MAPE	25.42 ± 0.80 ^b^	0.459 ± 0.002 ^d^	0.832 ± 0.003 ^c^	0.551 ± 0.001 ^d^
HDPE40AF8MAPE	31.12 ± 0.08 ^de^	0.451 ± 0.001 ^c^	0.873 ± 0.002 ^d^	0.517 ± 0.001 ^c^
HDPE50AF8MAPE	32.01 ± 0.66 ^e^	0.454 ± 0.001 ^c^	0.902 ± 0.0019 ^e^	0.503 ± 0.002 ^a^
BioPE20AF8MAPE	38.13 ± 0.52 ^f^	0.435 ± 0.001 ^a^	0.741 ± 0.002 ^a^	0.588 ± 0.001 ^f^
BioPE30AF8MAPE	29.90 ± 0.43 ^d^	0.447 ± 0.001 ^b^	0.812 ± 0.001 ^g b^	0.551 ± 0.001 ^d^
BioPE40AF8MAPE	28.17 ± 0.51 ^c^	0.453 ± 0.001 ^c^	0.876 ± 0.001 ^b d^	0.518 ± 0.001 ^c^
BioPE50AF8MAPE	27.64 ± 0.54 ^c^	0.459 ± 0.001 ^d^	0.906 ± 0.001 ^e^	0.506 ± 0.001 ^b^

Different letters, a, b, c, d, e, and f, represent the statistical differences (ANOVA, *p* < 0.05) between the properties of the materials.

**Table 3:** Influence of AF content over the tensile properties of 8wt.% coupled composites.

Composite	$E_{t}^{F}$ (GPa)	*α*_r_ (°)	*α*_s_ (°)	*α*_t_ (°)
HDPE20AF8MAPE	21.60 ± 0.12 ^a^	27.3°	36.1°	38.4°
HDPE30AF8MAPE	26.24 ± 0.31 ^b^	28.3°	37.3°	39.7°
HDPE40AF8MAPE	33.40 ± 0.68 ^d^	29.7°	39.1°	41.6°
HDPE50AF8MAPE	34.88 ± 0.78 ^d^	30.3°	39.9°	42.4°
HDPE mean		28.9° ± 1.4	38.1° ± 1.7	40.5° ± 1.8
BioPE20AF8MAPE	43.54 ± 1.94 ^e^	26.7°	35.3°	37.6°
BioPE30AF8MAPE	33.02 ± 1.06 ^d^	28.3°	37.3°	39.7°
BioPE40AF8MAPE	29.87 ± 0.09 ^c^	29.7°	39.1°	41.6°
BioPE50AF8MAPE	29.15 ± 0.97 ^c^	30.2°	39.7°	42.2°
BioPE mean		28.7° ± 1.6	37.9° ± 2.0	40.25° ± 2.1

Different letters, a, b, c, d, and e, represent the statistical difference (ANOVA, *p* < 0.05) between the properties of the materials.

## Data Availability

Data is available from the corresponding author on reasonable request.
